# Retraction Note: Effects of bilateral Pecto-intercostal Fascial Block for perioperative pain management in patients undergoing open cardiac surgery: a prospective randomized study

**DOI:** 10.1186/s12871-024-02839-5

**Published:** 2024-11-29

**Authors:** Yang Zhang, Haixia Gong, Biming Zhan, Shibiao Chen

**Affiliations:** 1 Department of Anesthesiology, First Affiliated Hospital of Nanchang University, 17 Yong wai zheng Street, Nanchang, 330006 Jiangxi China; 2 Department of cardiology, The second Affiliated Hospital of Nanchang University, NO.1 minde Street, Nanchang, 330006 Jiangxi China

**Retraction Note: BMC Anesthesiology (2021) 21:175**

10.1186/s12871-021-01391-w

The Editor has retracted this article. After publication, concerns were raised regarding the reporting of this randomised controlled study. An investigation has found that there are significant differences between the age range in the inclusion criteria stated in the trial registration record and those reported in this article. The authors have been unable to sufficiently justify these differences. Therefore, the Editor no longer has confidence in the results and conclusions presented.

None of the authors have responded to correspondence from the Editor about this retraction.

